# The Synthesis of Novel Glucosylamide Organosilicon Quaternary Ammonium Salts and Long-Lasting Modification of Different Materials

**DOI:** 10.3390/molecules30193934

**Published:** 2025-10-01

**Authors:** Xiangji Meng, Yunkai Wang, Jingru Wang, Lifei Zhi, Linfei Li, Xiaoming Li, Chan Wu, Rui Jin, Ziyong Ma, Zhiwang Han, Xudong Liu

**Affiliations:** 1 College of Chemical Engineering and Technology, Taiyuan University of Science and Technology, Taiyuan 030024, China; s202321111068@stu.tyust.edu.cn (X.M.); wyk990306@163.com (Y.W.); s202321111083@stu.tyust.edu.cn (J.W.); lilinfei@tyust.edu.cn (L.L.); xiaomingli@tyust.edu.cn (X.L.); 202421020224@stu.tyust.edu.cn (C.W.); hahzw@tyust.edu.cn (Z.H.); 2 Jiangsu Wanqi Biotechnology Co., Ltd., Hai’an 226600, China; jinrui@wanqicn.net; 3 Hai’an R&D Center of High-End Equipment and Rail Transit, Taiyuan University of Science and Technology, Hai’an 226600, China; zyma_sc@tyust.edu.cn; 4 Taiyuan Hengdeyuan Animal Health Care Technology R&D Co., Ltd., Taiyuan 030024, China; 13835179079@163.com

**Keywords:** surface modification, organosilicon quaternary ammonium salts, surface activity, foam properties, spreading performance

## Abstract

Using renewable D-gluconic acid δ-lactone as the starting material, two novel glucosamide-based organosilicon quaternary ammonium surfactants (**2/3SiDDGPBH**) were synthesized through an environmentally friendly three-step process involving amidation, hydrophobic modification, and quaternization. Comprehensive characterization demonstrated their exceptional performance: surface tension reduction to 33.4 mN/m (**2SiDDGPBH**) and 33.64 mN/m (**3SiDDGPBH**), uniform spherical micelles (1–10 nm and 30–100 nm) were formed, and outstanding foam properties with **3SiDDGPBH** developed, showing superior foamability and stability. Material modification tests on polymethyl methacrylate (PMMA) plates, mature acacia leaves, oilpaper, vegetable-tanned top-grain leather, and melamine-formaldehyde resin (MFR) faced with plywood revealed excellent spreading performance and durability, particularly for **3SiDDGPBH**-treated MFR plywood, which maintained excellent spreading performance even after 80 washing cycles. Scanning electron microscopy (SEM) analysis confirmed that the Si wt% of MFR plywood treated with **2/3SiDDGPBH** and scrubbed MFR plywood both exhibited a significant increase, and the **3SiDDGPBH**-treated MFR plywood demonstrated superior bonding properties. These surfactants combine low surface tension, excellent foaming properties, and outstanding spreading performance, demonstrating broad application prospects in fields such as pesticide adjuvants, industrial and household cleaning agents, cosmetics, oilfield extraction, textile printing and dyeing, and functional coatings.

## 1. Introduction

In the aftermath of the “14th Five-Year Plan” for the Development of Raw Material Industry in 2021, the chemical industry has undergone a pronounced transformation, pivoting towards high-end, green, and intelligent production in alignment with the national “dual carbon” objective. This strategic initiative provides explicit directives for the advancement of green, durable, and environmentally friendly surfactants. The development of green, long-lasting, and environmentally friendly surfactants has provided clear guidelines, and surfactant modification materials have also received extensive attention [1,2,3]. The term “long-lasting surfactant” refers to the surfactant’s ability to maintain its efficacy over an extended period during its application, which remains largely unaffected by environmental factors and conditions of use. The merits of long-lasting surfactants are manifold. The prolongation of the action time following a wipe, wash, or mechanical rub is conducive to the maintenance of optimal interfacial activity, thereby reducing waste and the overall cost. For instance, the addition of surfactants to shampoos has been demonstrated to enhance the stability of the resulting foam [4]. Furthermore, the incorporation of these surfactants has been shown to facilitate the effective distribution of pesticides, thereby ensuring their adherence during rainfall [5,6]. In addition, the utilization of surfactants has been identified as a means to minimize the frequency of replenishment or repeated processing, thereby enhancing ease of use and reducing resource consumption, in accordance with the principles of sustainable development. In the context of oil extraction, the utilization of surfactants has been demonstrated to be an effective strategy for reducing interfacial tension at the oil-water interface, thereby enhancing the efficiency of extraction processes over an extended period [7]. This approach has the advantage of prolonging the lifespan of surfactants, leading to a significant reduction in the frequency of extraction operations and, consequently, a decrease in environmental impact. These advantages confer upon them a unique set of benefits across multiple disciplines.

**Figure 1:**
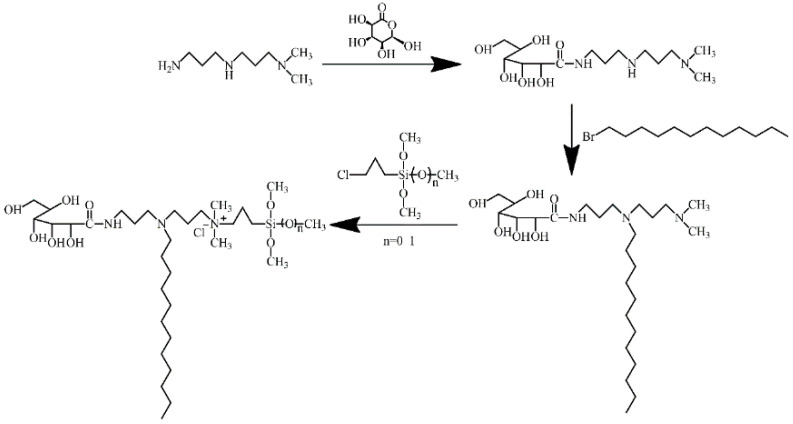
Synthesis route of mixed double-stranded glucosylamide organosilicone quaternary ammonium surfactant.

Surfactants of a conventional nature are generally confronted with the predicament of inadequate longevity in practical applications. This is predominantly associated with the molecular adsorption mode. Conventional surfactants are predominantly bound to the material surface through weak physical adsorption, a phenomenon that is contingent on intermolecular forces such as van der Waals force and hydrogen bonding. This binding process is characterized by low bond energy and pronounced reversibility. When subjected to external wiping or mechanical friction, the adsorbed molecular layer is susceptible to destruction, leading to desorption and subsequent rapid decay of surface activity. This limitation significantly restricts the application of surfactants in fields requiring long-term maintenance of surface performance. Consequently, the development of surfactant systems with long-term stability is of significant scientific and engineering value, in addition to having broad market prospects and urgent application needs [8,9,10,11,12]. In this paper, two glucosylamide organosilicon quaternary ammonium salts (**2/3SiDDGPBH**) were synthesized through a series of reactions and modifications and were demonstrated to exhibit remarkable durability. First, the amidation reaction of δ-lactone of D-gluconate with N, N-dimethyldipropenyltriamine is examined. Subsequently followed by a substitution reaction with dodecyl bromide. The final step in the synthesis was a quaternization reaction with γ-chloropropylmethyldimethoxysilane/γ-chloropropyltrimethoxysilane (Figure 1). The structures of the products were characterized by Fourier infrared spectroscopy (FT-IR) and nuclear magnetic resonance (NMR), and the structures of the precursors, intermediates, and products were determined. Furthermore, we employed a series of industrially relevant substrates with distinct surface characteristics to rigorously evaluate the durability and spreading performance of the synthesized **2/3SiDDGPBH**.

Polymethylmethacrylate (PMMA): Selected for its high transparency and versatility, PMMA is an irreplaceable material in modern industries such as automotive, medical devices, and optical applications [13,14,15]. However, its inert, non-polar surface dominated by ester and methyl groups presents a significant challenge for durable modification, representing a stringent test for surfactant anchoring capability.

Mature acacia leaves: Chosen as a model for plant foliage, enhancing the wettability and retention of agrochemicals on leaf surfaces is crucial for improving pesticide efficiency and reducing environmental loss due to rainwater scouring [16]. Furthermore, hydrophilic modification can enhance the potential of leaves as natural adsorbents for environmental remediation [17].

Oiled paper: This material finds extensive use in packaging, agriculture, and protective layers. Hydrophilic treatment can significantly expand its functionality, for instance, in medical dressings for improved liquid absorption or in the conservation of cultural artifacts (e.g., ancient books and paintings) by enhancing the penetration of restoration adhesives [18].

Vegetable-tanned cowhide: The inherent hydrophobicity of natural collagen limits the comfort and functionality of leather products. Imparting a durable hydrophilic finish, coupled with the antibacterial properties of quaternary ammonium salts, can unlock new applications in high-comfort footwear, outdoor apparel, and hygienic products [19,20].

Melamine-formaldehyde resin plywood (MFR plywood): While highly valued in high-end furniture and interior decoration for its durability and abrasion resistance [21,22], its highly hydrophobic surface limits use in environments requiring easy cleaning and hygiene. Durable hydrophilic modification aims to enable its application in medical settings and public health scenarios where easy cleanability and biosafety are paramount [23].

The spreading properties and durability of **2/3SiDDGPBH** on these five representative substrates were systematically investigated through contact angle measurements and washout resistance experiments, the results of which are presented and discussed in the following section.

## 2. Results and Discussion

### 2.1. Structural Identification

The raw materials, precursors, and products of the reaction were characterized by FT-IR, ^1^H NMR, and ^13^C NMR spectroscopy.

**Figure 2:**
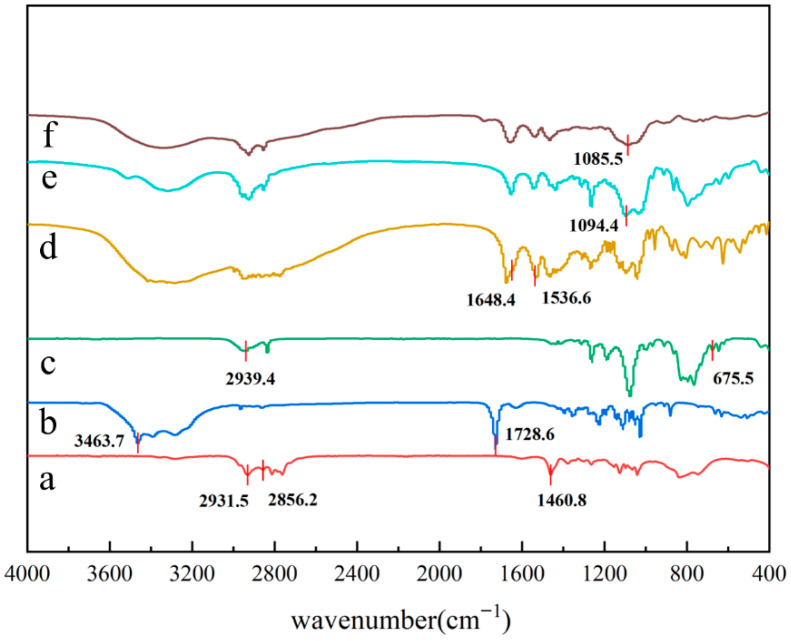
FT-IR infrared spectra (a) N, N-dimethyldipropylamine (b) gluconolactone (c) γ- chloropropylmethyldimethoxysilane (d) **DDGPD** (e) **2SiDDGPBH** (f) **3SiDDGPBH**.

As illustrated in Figure 2, the -CH_3_ and -CH_2_ stretching vibrational absorption peaks are located at 2931.5 cm^−1^ and 2856.2 cm^−1^, respectively. The absorption peak at 1460.8 cm^−1^ is identified as the characteristic peak of the shear vibration of -NH_2_ in N,N-dimethyldipropenyltriamine. The -OH stretching vibration absorption peak at 3463.7 cm^−1^ and the strong absorption peak at 1728.6 cm^−1^ are characteristic absorption peaks of the stretching vibration of C=O in D-gluconate δ-lactone. The two strong absorption peaks at 1536.6 cm^−1^ and 1648.4 cm^−1^ are characteristic peaks of the stretching vibration of the C=O bond in the amide bond and the bending vibration of the N-H peaks, respectively. These results indicate the presence of an amide bond and the generation of **DDGPD**. The characteristic absorption peaks of quaternary ammonium salt, located at 1085.5 cm^−1^ and 1094.4 cm^−1^, serve as indicative markers of its successful synthesis. The C-Cl stretching vibration peak at 675.5 cm^−1^, which is characteristic of the 3-chloropropylmethyldimethoxysilane product, exhibited a notable absence in the present study. Preliminary evidence suggests the synthesis of products.

^1^H-NMR(DMSO, ppm): δ: 0.83 (t, 3H, N(CH_2_)_11_C**H**_3_), 1.04 (t, 2H, SiC**H**_2_), 1.22 (s, 20H, NCH2(C**H**_2_)_10_CH_3_), 1.78 (t, 6H, NCH_2_(C**H**_2_)_2_, N^+^CH_2_C**H**_2_), 2.41 (s, 2H, NC**H**_2_), 2.55 (s, 2H, NC**H**_2_), 2.92 (t, 2H, NC**H**_2_), 3.04 (t, 6H, NCH_2_CH_2_C**H**_2_NH, NCH_2_CH_2_C**H**_2_N^+^, N^+^C**H**_2_), 3.15 (s, 6H, N^+^(C**H**_3_)_2_, 3.28 (s, 1H, COCHOHCHOHC**H**OHCHOHCH_2_OH), 3.41 (m, 9H, SiO(C**H**_3_)_3_), 3.43 (m, 3H, COCHOHCHOHCHOHC**H**OHC**H**_2_OH), 3.74 (s, 2H, COCHOHC**H**OHCHOHCHOHCH_2_O**H**), 3.91 (s, 2H, COC**H**OHCHOHCHO**H**CHOHCH_2_OH), 4.02 (t, 2H, COCHOHCHO**H**CHOHCHO**H**CH_2_OH), 5.30 (s, 1H, COCHO**H**CHOHCHOHCHOHCH_2_OH), 7.95 (t, 1H, CON**H**).

^13^C-NMR(DMSO, ppm): 5.85 (N^+^CH_2_**C**H_2_), 13.98 (N(CH_2_)_11_**C**H_3_, 18.56(Si**C**H_2_), 22.15 (NCH_2_**C**H_2_CH_2_N^+^, N(CH_2_)_10_**C**H_2_, 25.88 (NCH_2_CH_2_**C**H_2_), 28.62 (NCH_2_**C**H_2_), 28.78 (N(CH_2_)_3_**C**H_2_**C**H_2_), 29.10 (N(CH_2_)_3_CH_2_CH_2_(**C**H_2_)_5_), 31.36 (NCH_2_**C**H_2_CH_2_NH), 39.52 (N**C**H_2_CH_2_CH_2_NH), 39.94 (NCH_2_CH_2_**C**H_2_NH), 48.61 (SiO(**C**H_3_)_3_, 50.12 (N^+^(**C**H_3_)_2_), 55.06 (N**C**H_2_CH_2_CH_2_N^+^), 56.07 (N**C**H_2_(CH_2_)_11_CH_3_), 63.27 (NCH_2_CH_2_**C**H_2_N^+^), 63.68 (COCHOHCHOHCHOHCHOH**C**H_2_OH), 70.16 (N^+^**C**H_2_), 71.45 (COCHOHCHOH**C**HOH**C**HOHCH_2_OH), 72.25 (COCHOH**C**HOHCHOHCHOHCH_2_OH), 73.70 (CO**C**HOHCHOHCHOHCHOHCH_2_OH), 173.37 (**C**ONH).

**Figure 3:**
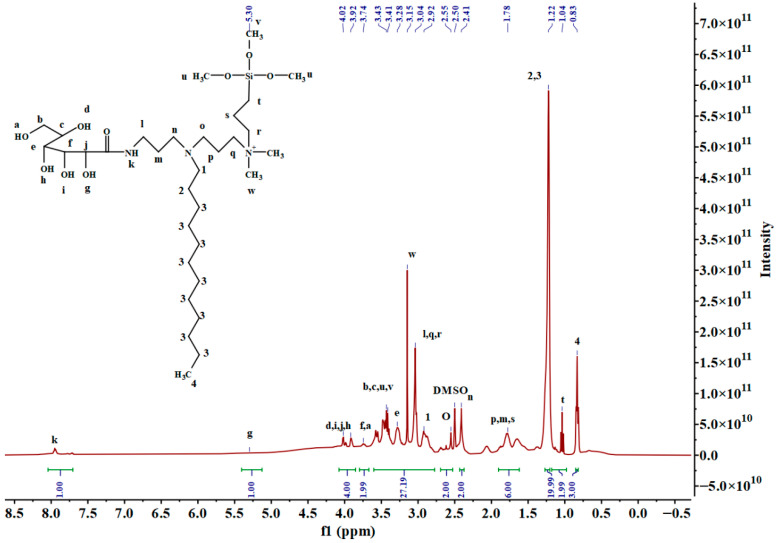
^1^H-NMR spectra of **3SiDDGPBH**. Lowercase letters denote the specific H atoms in the product discussed in the NMR analysis.

**Figure 4:**
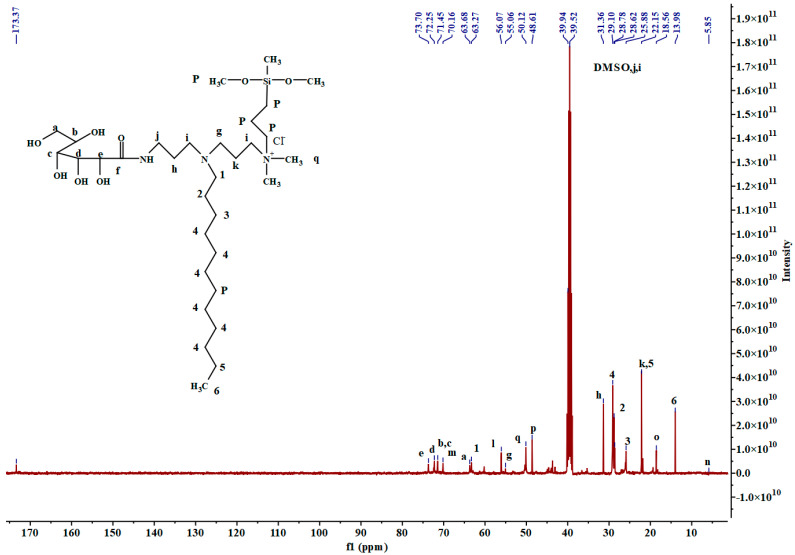
^13^C-NMR spectra of **3SiDDGPBH**. Lowercase letters denote the specific C atoms in the product discussed in the NMR analysis.

As illustrated in Figure 3, the ^1^H NMR spectrum of **3SiDDGPBH** is displayed, while Figure 4 depicts the ^13^C NMR spectrum of the same compound. (Comprehensive data pertaining to the ^1^H NMR and ^13^C NMR spectra of the precursor, intermediate, and **2SiDDGPBH** can be found in the Supplementary Materials). In the ^1^H NMR spectrum (Figure 3), the peak at 8.0 ppm corresponds to the characteristic peak of an amide bond, indicating the synthesis of a precursor and an intermediate. In addition, the characteristic peaks of chain sub-methylene (-CH_2_-) and chain-terminal methylene (-CH_3_) appear at 0.9 ppm to 1.3 ppm. The target products were successfully synthesized. Their structures were confirmed by FT-IR, ^1^H NMR, and ^13^C NMR spectroscopy. This synthetic route is concise and efficient. It generates no waste and meets the requirements for green industrial production.

### 2.2. Surface Activity

**Figure 5:**
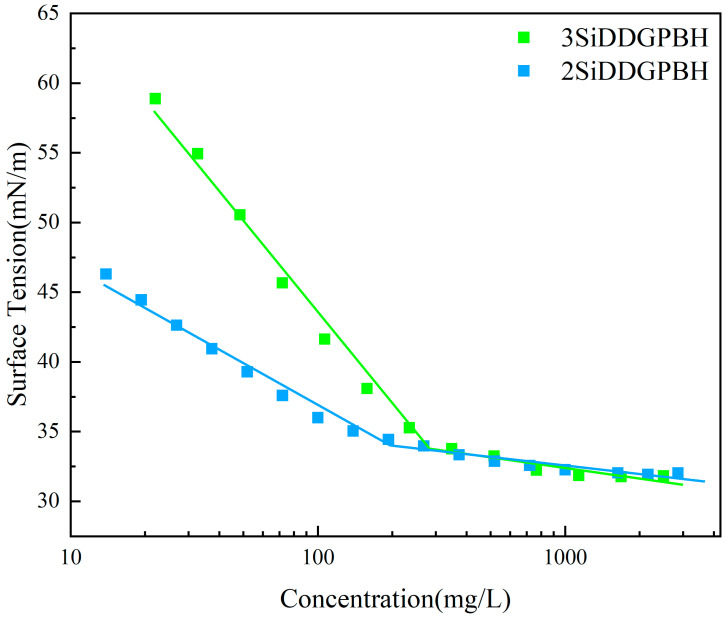
Surface tension of **2/3SiDDGPBH** in an aqueous solution as a function of concentration.

In this study, the surface tension of **2/3SiDDGPBH** was determined by the platinum ring method at 25 °C ± 0.1 °C. The experimental findings demonstrated that the surface tension of the solution exhibited a linear decrease with an increase in surfactant concentration until the critical micelle concentration (CMC) was attained. This phenomenon can be attributed to the gradual formation of a regular arrangement of surfactant molecules at the interface. Upon reaching the critical micelle concentration (CMC) point, the curve undergoes a substantial change, and subsequently, the surface tension of the solution exhibits a reduced response to the equilibrium surface tension curve (γ-logc curve) as the surfactant concentration continues to be increased (Figure 5). Surface adsorption parameters were determined using the simplified form of the Gibbs adsorption isotherm (Equations (1) and (2)). The standard Gibbs free energies of adsorption and micellization in aqueous solution ($\Delta G_{ads}^{ϴ}$ and $\Delta G_{mic}^{ϴ}$) were calculated using Equations (3) and (4), respectively [24,25,26].

(1) $$\Gamma_{max}=-\frac{1}{2.303nRT}(\frac{\partial \gamma }{\partial logc}{)}_{T}$$

(2) $$A_{min}=\frac{1}{N_{A}\Gamma_{max}}$$

(3) $$\Delta G_{ads}^{ϴ}=nRTln\left(\frac{CMC}{\alpha }\right)-6.022\pi A_{min}$$

(4) $$\Delta G_{mic}^{ϴ}=nRTln\left(\frac{CMC}{\alpha }\right)$$

For the cationic surfactant used in this study, *n* was considered to be 2. Here *α* is the molar concentration of water (55.3 at 25 °C), *R* is the molar gas constant (8.314 J·mol^−1^·K^−1^), *T* denotes the absolute temperature (K), *N_A_* is the Avogadro constant (6.022 × 10^23^ mol^−1^), *π* = (*γ*_0_ − *γ_CMC_*) is the surface tension in the saturated region of the surface, and *γ*_0_ is the surface tension of water (72 mN/m). At a temperature of 25 °C, the maximum surface excess concentration (mol/m^2^) is denoted by “*Γ_max_*,” and the calculated values of the surface activity characteristic parameters are summarized in Table 1.

As illustrated in Figure 5, **2/3SiDDGPBH** exhibits a pronounced non-polarity, attributable to the incorporation of a protracted carbon chain configuration. At adsorption saturation on the surface, the surfactant’s intermolecular forces undergo a substantial diminution, concomitant with a significant reduction in the critical micelle concentration (*CMC*). Furthermore, the experimental findings demonstrated that both $\Delta G_{ads}^{ϴ}$ and $\Delta G_{mic}^{ϴ}$ were negative, thereby substantiating the spontaneous nature of the adsorption and micellization processes. The magnitude of the free energy change (∣$\Delta G_{ads}^{ϴ}$∣ > ∣$\Delta G_{mic}^{ϴ}$∣) indicates that **2/3SiDDGPBH** is more prone to undergoing adsorption behavior than micellization in an aqueous solution [27,28,29]. It is noteworthy that the surface tension of **2/3SiDDGPBH** continued to decrease slowly with increasing concentration, which may be related to the multiple adsorption mechanism. At high concentrations, surfactant molecules may have continued to rearrange at the interface or formed additional adsorption layers, further reducing the surface tension [30].

**Figure 6:**
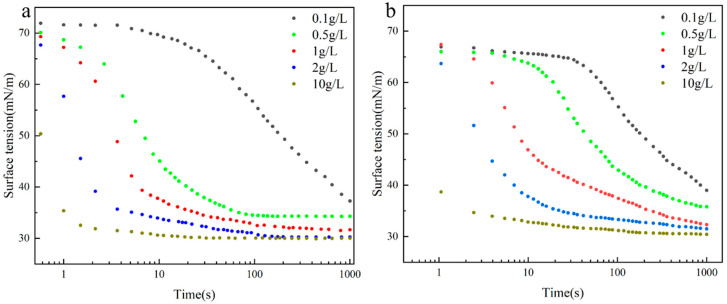
The relationship between the dynamic surface tension of **2/3SiDDGPBH**. (**a**) **2SiDDGPBH**, (**b**) **3SiDDGPBH**.

As illustrated in Figure 6, in contrast to equilibrium surface tension, dynamic surface tension is defined as the relationship between surface tension and time, which reflects the adsorption rate of surfactant molecules transferred from the bulk phase to the interface. According to Rosen et al., the dynamic surface tension profile can be subdivided into four zones: the induction zone, the rapid decline zone, the meso-equilibrium zone, and the equilibrium zone [31,32,33]. The dynamic surface tension profiles of all samples exhibited a decreasing trend with increasing concentration, and the magnitude and rate of decrease increased. It is important to note that, due to instrumental limitations, the data for the induced and equilibrium zones for some samples could not be obtained. However, the results clearly showed that the induction zone gradually narrowed as concentration increased, while the rapid decay zone emerged earlier. This behavior can be explained by the enhanced diffusion of surfactant molecules under steeper concentration gradients between the bulk phase and the adsorption layer. As a result, the kinetics of surface tension reduction were significantly accelerated.

At the concentration of 0.1 g/L, the diffusion rate remained slow due to the limited concentration gradient, whereas at all higher concentrations, the rapid decay zone emerged earlier with a significantly accelerated decline rate. This behavior is primarily attributed to the enhanced hydrophobicity resulting from the incorporation of the long carbon chain, which substantially reduces the Gibbs free energy of adsorption, thereby providing stronger thermodynamic driving force for interfacial adsorption. Meanwhile, the increased hydrophobicity promotes tighter molecular packing at the interface, exposing more hydrophobic -CH_3_ and enhancing van der Waals interactions. These effects collectively strengthen the molecular-interface interactions and synergistically accelerate the adsorption kinetics, ultimately leading to a more rapid decline in dynamic surface tension.

### 2.3. Aggregation Behavior in an Aqueous Solution

**Figure 7:**
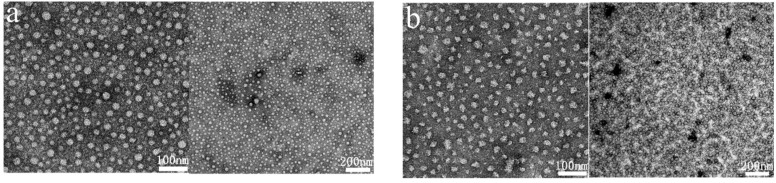
Transmission electron micrographs of different samples at 10 g/L concentration. (**a**) **2SiDDGPBH**, (**b**) **3SiDDGPBH**.

**Figure 8:**
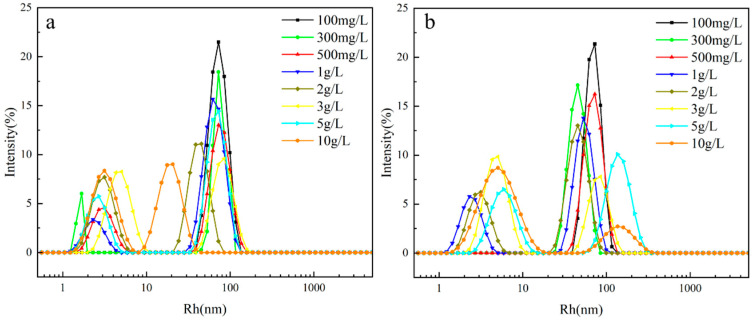
The intensity-weighted size distributions at different concentrations (**a**) **2SiDDGPBH**, (**b**) **3SiDDGPBH**.

As illustrated in Figure 7, the microscopic morphology observed by transmission electron microscopy (TEM) revealed that **2SiDDGPBH** displays a spherical micellar structure that is uniformly dispersed, with a smooth surface and narrow size distribution. This suggests that the synergistic interaction between its long carbon chains (C12) and siloxane groups (Si-O-Si) promotes the orderly intermolecular stacking. The long carbon chains form a dense inner core through hydrophobic interactions, while the siloxane shells achieve interfacial stabilization through hydrogen bonding with glycosyl hydrophilic groups (-OH). The particle size distribution of **2/3SiDDGPBH** was further analyzed by dynamic light scattering (DLS), and the results indicated a bimodal distribution with nm-scale aggregate sizes (Figure 8). The DLS intensity distribution of **2/3SiDDGPBH** exhibits a sharp and narrow main peak with limited shoulder broadening, indicating the formation of a compact micellar core driven by strong hydrophobic interactions, which suppresses dynamic rearrangement of molecular chains and reduces aggregation tendency. It is noteworthy that the bimodal size distribution of **3SiDDGPBH** is slightly larger overall than that of **2SiDDGPBH**, consistent with the critical micelle concentration measurements. This bimodal profile suggests a tendency of the molecules to form diverse self-assembled morphologies in solution.

### 2.4. Foam Properties

**Figure 9:**
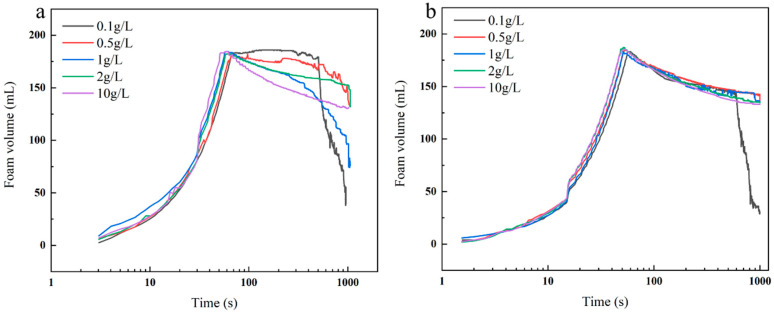
Changes of **2/3SiDDGPBH** foam height over time at different concentrations. (**a**) **2SiDDGPBH**, (**b**) **3SiDDGPBH**.

**Figure 10:**
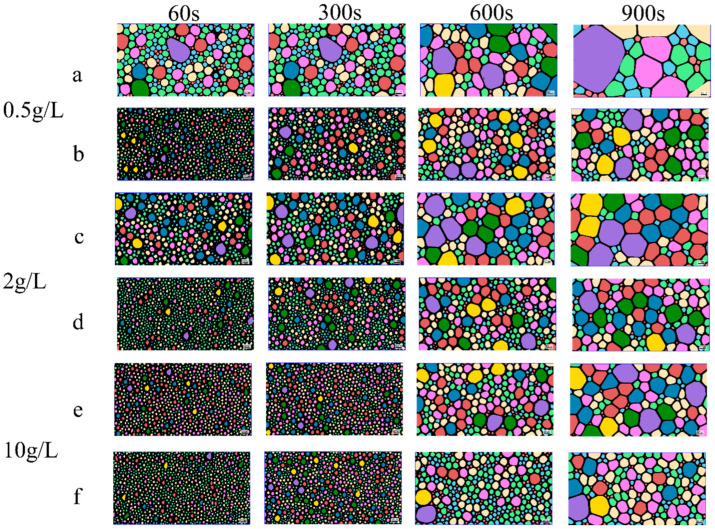
Foam photographs and distribution of **2/3SiDDGPBH** at different concentrations. (**a**) 0.5 g/L **2SiDDGPBH**, (**b**) 0.5 g/L **3SiDDGPBH**, (**c**) 2 g/L **2SiDDGPBH**, (**d**) 2 g/L **3SiDDGPBH**, (**e**) 10 g/L **2SiDDGPBH**, (**f**) 10 g/L **3SiDDGPBH**.

**Figure 11:**
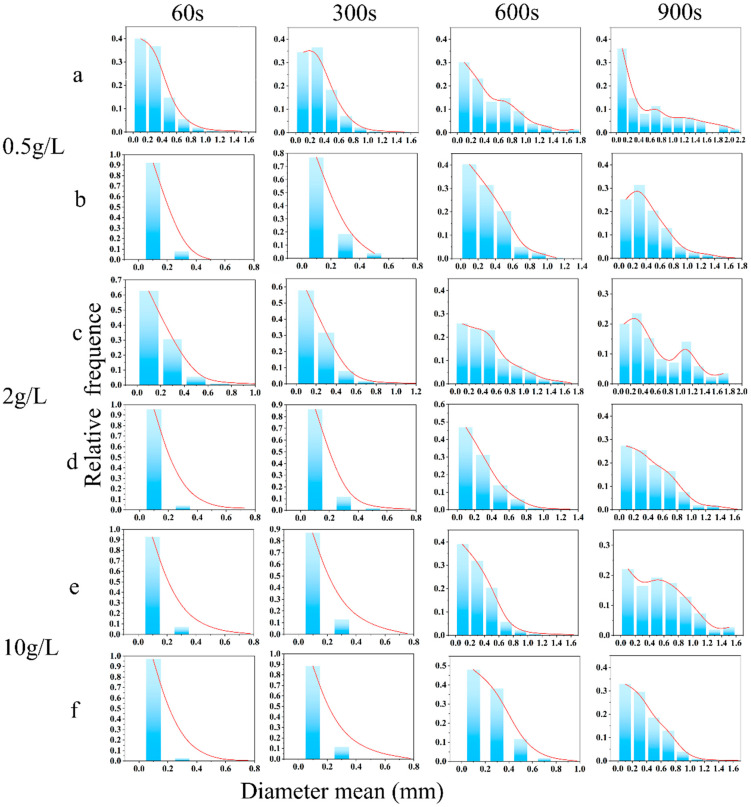
Foam size distribution histogram of **2/3SiDDGPBH** at different concentrations. (**a**) 0.5 g/L **2SiDDGPBH**, (**b**) 0.5 g/L **3SiDDGPBH,** (**c**) 2 g/L **2SiDDGPBH**, (**d**) 2 g/L **3SiDDGPBH**, (**e**) 10 g/L **2SiDDGPBH**, (**f**) 10 g/L **3SiDDGPBH**.

The comprehensive evaluation of foam stability, as shown in Figure 9, Figure 10 and Figure 11, demonstrated the superior performance of **3SiDDGPBH** over **2SiDDGPBH**. Quantitatively, **3SiDDGPBH** maintained a stable foam volume of 140–150 mL over 1000 s at concentrations ≥0.5 g/L, whereas **2SiDDGPBH** exhibited rapid collapse. Morphologically, the foam generated by **3SiDDGPBH** was characterized by a higher density of smaller, more uniform bubbles and a significantly slower rate of coalescence over time, indicating a more persistent and finer foam architecture.

This notable enhancement in foam stability is fundamentally governed by the Gibbs-Marangoni effect, a self-healing mechanism whose efficiency is intrinsically linked to the molecular structure of the surfactant. The effect is initiated by local film thinning, which creates a surface tension gradient that drives surfactant migration and liquid flow to repair the weak spot. The critical parameter here is the interfacial diffusion coefficient. At the same concentration, the larger molecular structure of **3SiDDGPBH**, specifically the presence of an additional methoxy group which imposes greater steric hindrance at the air-water interface, confers a lower interfacial diffusion coefficient compared to **2SiDDGPBH**. This slower diffusion is advantageous as it sustains the surface tension gradient for a longer duration, thereby prolonging the Marangoni-driven healing action and effectively preventing bubble coalescence.

Furthermore, the present study revealed a notable phenomenon: the foam stability of both compounds exhibited a declining trend with increasing surfactant concentration. This observed behavior can be explained by the competitive balance mechanism between interfacial adsorption and bulk micellization processes. At elevated concentrations, this essential repair mechanism is severely compromised by micellization. The formation of bulk micelles depletes the reservoir of free surfactant monomers available for rapid adsorption. Consequently, the replenishment of surfactants to the interface is kinetically hindered, diminishing the Marangoni restoring flow and ultimately leading to foam instability.

The foam of the product has been shown to possess capacity-enhancing, bubble-stabilizing, and dirt removal efficiency. Consequently, it is anticipated that the product will find application in a variety of fields, including washing, firefighting, flotation, and food processing. These applications are expected to enhance cleaning efficiency and optimize industrial processes [34,35].

### 2.5. Spreading Performance

Surface spreadability is a critical index of material surface performance, with a direct impact on the material’s waterproof, anti-fouling, and self-cleaning capabilities. Contact angle is a frequently utilized metric for characterizing the surface spread ability of materials, with its magnitude reflecting the interaction strength between liquid and solid surfaces. The spreading properties of modified materials can be reflected by measuring the contact angle of droplets on modified low surface energy substrates polymethylmethacrylate (PMMA) boards, mature acacia leaves, oiled paper, vegetable-tanned cowhide, melamine-formaldehyde resin-finished plywood (MFR plywood) [36,37,38,39].

**Figure 12:**
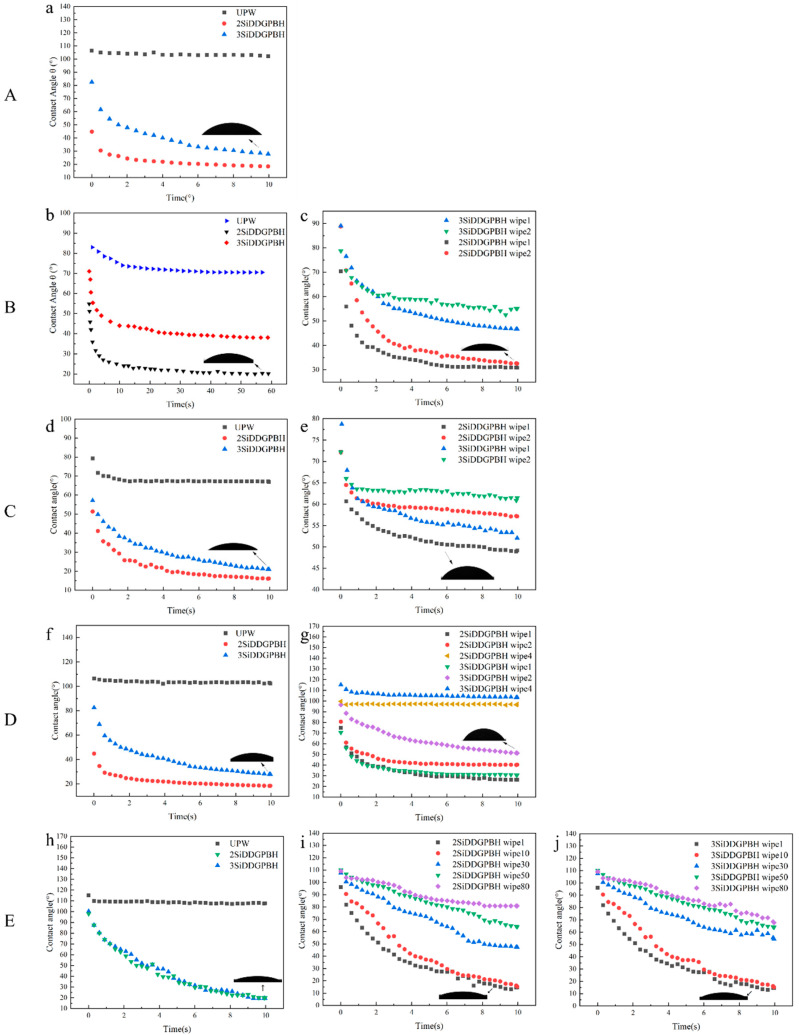
The contact angle of **2/3SiDDGPBH** on different infrastructure materials over time. (**A**) Polymethyl methacrylate (PMMA board) (**B**) Mature acacia leaves (**C**) Oiled paper (**D**) Vegetable tanned first layer of cowhide (**E**) Melamine-formaldehyde resin plywood (MFR plywood). (**a**) Contact angles of 2/3SiDDGPBH and UPW on a PMMA substrate (**b**) Contact angles of 2/3SiDDGPBH and UPW on a Mature acacia leaves substrate (**c**) UPW contact angles on mature acacia leaves after two wiping cycles (**d**) Contact angles of 2/3SiDDGPBH and UPW on an Oiled paper substrate (**e**) UPW contact angles on Oiled paper after two wiping cycles (**f**) Contact angles of 2/3SiDDGPBH and UPW on an Vegetable tanned first layer of cowhide substrate (**g**) UPW contact angles on Vegetable tanned first layer of cowhide after four wiping cycles (**h**) Contact angles of 2/3SiDDGPBH and UPW on a MFR plywood substrate (**i**) UPW contact angles on MFR plywood after eighty wiping cycles (**j**) UPW contact angles on MFR plywood after eighty wiping cycles.

As illustrated in Figure 12A, the contact angle of the distilled water blank sample of the unmodified PMMA plate was 100°, and the contact angle of the PMMA plate treated with **2SiDDGPBH** quickly decreased from the initial value of 55° to 20° and the **3SiDDGPBH** decreased to 30° within 10 s, indicating that the surface of the material changed from hydrophobic to hydrophilic. However, the contact angle of the washed PMMA plate was restored to the same as that of the blank sample.

As illustrated in Figure 12B, the mature acacia leaves exhibited a contact angle of 70° for the unmodified leaves distilled water blank sample. In contrast, the static water contact angle after **2/3SiDDGPBH** treatment demonstrated a significantly faster decrease, reaching 20° versus 38°, respectively, after equilibrium (Figure 12b). Both samples remained effective after two washes, especially the **2SiDDGPBH**-treated leaves, where the static water contact angle could still be reduced to 30°, providing a good spreading effect (Figure 12c).

As illustrated in Figure 12C, the contact angle of the oil paper was measured to be 70° with the distilled water blank sample. However, the contact angle of the oil paper treated with **2SiDDGPBH** rapidly decreased to 20° within 10 s, while **3SiDDGPBH** decreased to 30° within the same time (Figure 12d). This hypothesis was further substantiated by washout resistance experiments, wherein both modified systems exhibited durability, with the contact angle increasing to 52° and 57°, respectively, after two washouts (Figure 12e).

As illustrated in Figure 12D, a vegetable-tanned first layer cowhide with a contact angle of 102° is presented, representing the unmodified cowhide distilled water blank sample. The contact angle of the **2SiDDGPBH**-treated cowhide exhibited a rapid decrease, reaching 20° within 10 s, while the contact angle of the **3SiDDGPBH** decreased to 30° within the same period. This observation indicates a transition from a hydrophobic to a hydrophilic surface of the material (Figure 12f). This hypothesis was subsequently validated through the execution of rinsing resistance experiments, which demonstrated that both modified systems exhibited commendable durability. Specifically, the contact angle of the **2SiDDGPBH**-treated samples persisted at 40° even after two washes, thereby substantiating the initial hypothesis. The efficacy of the solution was maintained after four washes, as evidenced in Figure 12g.

As illustrated in Figure 12E, the MFR plywood exhibited a contact angle of 110° for the unmodified MFR plywood distilled water blank sample, while the contact angle of the **2/3SiDDGPBH**-treated MFR plywood demonstrated a rapid decrease, reducing from the initial value of 110° to 20° within 10 s. This observation signifies a transition from a hydrophobic to a hydrophilic surface state of the material, as depicted in Figure 12h. This hypothesis was further substantiated by washout resistance experiments, which demonstrated that both modified systems exhibited commendable durability. Specifically, the contact angle of the **2SiDDGPBH**-treated samples remained at 75° after 80 washouts, while that of the **3SiDDGPBH**-treated samples persisted at 65° after the same number of washouts (Figure 12i,j).

While all substrates showed excellent initial hydrophilicity, their durability upon washing varied significantly, depending on the substrate type. This study demonstrates that both **2SiDDGPBH** and **3SiDDGPBH** exhibit excellent immediate hydrophilic modification capabilities on five substrates (PMMA, mature acacia leaves, oiled paper, vegetable-tanned cowhide, and MFR plywood). The durability of the modification effect is closely related to substrate characteristics: on substrates with active sites (e.g., leaves, oiled paper, leather, MFR plywood), covalent bonding formed between silane groups and surface groups (e.g., amino groups) results in outstanding wash resistance. In contrast, on inert PMMA surfaces—which lack active reaction sites such as hydroxyl or amino groups and are primarily composed of ester (-COOCH_3_) and methyl (-CH_3_) groups—the modification layer relies solely on physical adsorption and is, therefore, easily removed by washing [40,41,42].

**Figure 13:**
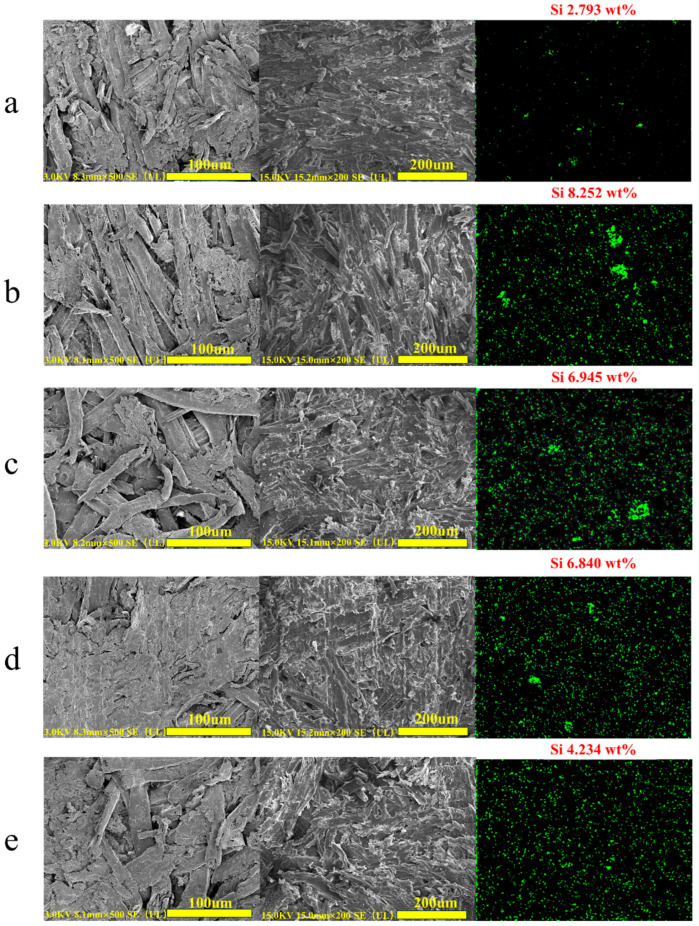
SEM images of **2/3SiDDGPBH** MFR plywood under different treatments (**a**) Untreated MFR plywood (**b**) MFR plywood treated with 2 g/L 3SiDDGPBH (**c**) MRR plywood treated with 2 g/L **2SiDDGPBH** (**d**) **3SiDDGPBH** MFR plywood with 80 scrubs (**e**) **2SiDDGPBH** MFR plywood after 80 scrubs.

To gain deeper insights into the interfacial bonding mechanism responsible for the remarkable durability observed on MFR plywood, we conducted detailed microscopic characterization using scanning electron microscopy (SEM) and energy-dispersive X-ray spectroscopy (EDS). SEM tests were conducted on untreated MFR plywood, MFR plywood treated with **2/3SiDDGPBH**, and MFR plywood after 80 scrubs. The results of these experiments are illustrated in Figure 13. The structural morphology of MFR plywood under the three different treatments did not show any significant changes. This indicates that **2/3SiDDGPBH** did not have a significant effect on the microstructure of MFR plywood and had a certain protective effect on MFR plywood. As illustrated in Figure 13b, the MFR plywood treated with **3SiDDGPBH** exhibited a significant increase in Si wt%, rising from 2.793% to 8.252%. This observation is in direct contrast to the untreated MFR plywood depicted in Figure 13a, which exhibited a comparatively lower Si wt% of 2.793%. After the scrubbing experiment, while the Si wt% of **3SiDDGPBH** diminished to 6.840%, it nevertheless exceeded that of the untreated MFR plywood, aligning with the modification pattern of the contact angle of MFR plywood (Figure 13d). Infrared spectroscopy was utilized to ascertain the spectral characteristics of unmodified MFR plywood. The analysis revealed the presence of stretching vibration peaks belonging to the amino group, with a frequency range of 3300 cm^−1^ to 3500 cm^−1^. Additionally, the spectrum exhibited C=N stretching vibration peaks of the triazine ring at 1650 cm^−1^ and bending vibration peaks of the amino group at 1500 cm^−1^. The derivatized ether bond of the cured melamine-formaldehyde resin was identified at 1200 cm^−1^ (-CH_2_-O-CH_2_-), thereby confirming the abundance of amino groups present on the surface of the material [43]. It is evident that **2/3SiDDGPBH** continues to demonstrate a satisfactory spreading effect on MFR plywood, even after multiple scrubbing passes. A comparative analysis of the SEM-EDS spectra of **2SiDDGPBH** and **3SiDDGPBH** indicates a higher silicon weight percentage (Si wt%) in **3SiDDGPBH** than in **2SiDDGPBH**, suggesting that **3SiDDGPBH** forms a stronger interfacial bond with the MFR plywood. This superior bonding performance originates from the following molecular-level mechanisms, the fundamental reason for this phenomenon can be attributed to: (1) R-Si-OCH_3_, a compound with silane properties, undergoes a methoxy demethanolization reaction, resulting in the formation of a silanol group (R-Si-OH). This process is followed by the dehydration and condensation of the amino group and silanol group on the surface of MFR plywood, leading to the formation of a strong covalent bond on the fiber surface. The presence of **3SiDDGPBH** facilitates the formation of additional silanol groups, thereby enhancing the strength of the bond. (2) The water-diluted R-Si-OH exhibits the capacity to achieve multi-effect synergy through electrostatic adsorption of positive and negative electrons, as well as weak interactions of van der Waals forces, when combined with MFR plywood.

## 3. Materials and Methods

### 3.1. Materials and Apparatus

D-gluconic acid δ-lactone (99%) from Aldrich (St. Louis, MO, USA), dodecyl bromide (chemically pure), N, N-dimethyldipropenyltriamine (99.15%), γ-chloropropylmethyldimethoxysilane (97%), γ-chloropropyltrimethoxysilane (97%), deuterated dimethyl sulfoxide (DMSO) (99.9% + 0.03% TMS), sodium phosphotungstate octadecahydrate Na_3_PO_4_-12WO_3_-xH_2_O (99%) were obtained from Shanghai Macklin Biochemical Co., Ltd. (Shanghai, China)

FT-IR spectrometer (VERTEX70), nuclear magnetic resonance instrument (AVANCE III) were from Bruker, (Bremen, Germany); fully automatic surface tension meter, model KRÜSS-Tensiio (Hamburg, Germany), fully automatic droplet analyzer (KRÜSS-DSA25S) were from Kreuz Scientific Instruments (Shanghai, China) Co., Ltd.; foam analyzer, model FOAMSCAN (Teclis, France), transmission electron microscope (JEM-F200) from Nippon Electron Corporation Co., Ltd. (Tokyo, Japan), nano particle size potentiometer (BeNano90Zeta) Dandong Baxter Instruments Co., Ltd. (Dandong, China).

### 3.2. Synthesis Methods of ***2/3SiDDGPBH***

Ethanol (55.23 g), N, N-dimethyldipropylenetriamine (25.94 g), and D-glucono-δ-lactone (29.3 g) were added to a 250 mL three-necked, round-bottomed flask equipped with a thermometer and a reflux condenser. The reaction was carried out under reflux conditions (temperature 82 °C) for a duration of 1 h. After standing and cooling at room temperature, the solvent was removed under reduced pressure using a rotary evaporator to obtain the crude product. The crude product was removed by washing with ether three times and dried under vacuum to obtain the precursor N-(3-((3-(dimethylamino) propyl) amino) propyl)-2,3,4,5,6-pentahydroxyacetamide (52.68 g) (**DDGPD**) as a light-yellow powder solid, yield rate is 95.37%.

Secondly, the precursor (52 g) and dodecyl bromide (38.76 g) were reacted in ethanol (55.23 g) under reflux at 82 °C for 5 h, using the same apparatus. Following a period of standing and cooling at room temperature, the solvent was removed under reduced pressure by a rotary evaporator. Thereafter, the solid was washed with ethyl ether on three occasions to yield a light-yellow powder. This solid was identified as intermediate N-(3-(3-((dimethylamino) propyl) (dodecyl) amino) propyl)-2,3,4,5,6-pentahydroxyacetamide (86.83 g) (**DDGPDH**), yield rate is 95.37%.

Finally, the intermediate (80 g), γ-chloropropylmethyldimethoxysilane (27.50 g)/γ-chloropropyltrimethoxysilane (29.80 g) ethanol (55.23 g) solvent was subjected to a reaction under reflux conditions (temperature 82 °C) for a duration of 20 h. The solution was subjected to a series of processes following its standing and cooling at room temperature. Initially, it was depressurized using a rotary evaporator to facilitate the removal of the solvent. Subsequently, the solution was washed with ether on three separate occasions. Finally, it was dried via vacuum to yield **2/3SiDDGPBH**. **2SiDDGPDH** is represented by the following formula: 3-(dimethoxy(methyl)silyl)-N-(3-(dodecyl(3-(2,3,4,5,6-pentahydroxyhexanamido) propyl) amino) propyl)-N, N-dimethylpropan-1-aminium chloride (101.45 g), yield rate is 95.37%. While **3SiDDGPDH** is represented by the following formula: 3-(dodecyl(3-(2,3,4,5,6-pentahydroxyhexanamido) propyl) amino)-N, N-dimethyl-N-(3-(trimethoxysilyl) propyl) propan-1-aminium chloride (104.49 g), yield rate is 95.37%.

### 3.3. Characterization of ***2/3SiDDGPBH***

The raw materials, N, N-dimethyldipropenyltriamine, gluconolactone, γ-chloropropylmethyldimethoxysilane, as well as their precursors and intermediates, were scanned in the wavelength range of 4000–400 cm^−1^ using the KBr compression method. The structures of the products were identified by observing the characteristic peaks of the generated infrared spectra. The synthesized products were subsequently measured using ^1^H-NMR and ^13^C-NMR, with the AVANCE III NMR spectrometer (Bremen, Germany) serving as the instrument for this purpose. Concurrently, the molecular structures of the synthesized compounds were analyzed through the examination of their NMR spectra.

### 3.4. Surface Tension

The equilibrium surface tension of **2/3SiDDGPBH** was measured using the platinum ring method with a KRÜSS-Tensiio Tensiometer (Hamburg, Germany), a fully automated surface tension meter. Initially, aqueous solutions of varying concentrations were meticulously prepared using deionized water as a solvent, ensuring the solutions were well-dissolved and homogeneous. Tests were conducted at a temperature of 25.0 ± 0.1 °C, and the instrument was calibrated using a standard solution prior to measurement.

### 3.5. Dynamic Surface Tension

In a temperature environment maintained at 25 °C ± 0.1 °C, the dynamic surface tension of **2/3SiDDGPBH** was measured using a fully automated drop-type measuring instrument. The experimental data was recorded.

### 3.6. Transmission Electron Microscopy

The structure of the **2/3SiDDGPBH** aggregates was analyzed using the negative staining method with a JEM-F200 field emission TEM (Tokyo, Japan). The samples were placed on a copper mesh for five minutes, blotted with filter paper, stained with 2 wt% sodium phosphotungstate for fifteen minutes, and stored at room temperature for a minimum of two hours prior to the experiment. The concentration of each experimental sample was 10 g/L.

### 3.7. Dynamic Light Scattering

Different concentrations of **2/3SiDDGPB**H solutions were prepared and left to stabilize and homogenize. Subsequently, 1–2 milliliters of the solution was transferred into a cuvette that was appropriate for the designated analysis at a temperature of 25 °C. The cuvette was then placed into the sample tank for the measurement process.

### 3.8. Foam Morphology Characterization

The experiment was executed employing the bulging gas method, utilizing a TECLIS Instruments (TECLIS) foam analyzer. The **2/3SiDDGPBH** solution was prepared in concentrations of 0.1, 0.5, 1, 2, and 10 g/L. The gas flow rate was set at a constant level of 150 milliliters per minute. The initial foam volume was determined to be 180 mL. The alterations in the quantity and configuration of the foams were meticulously observed for a duration of 1000 s. The foam was meticulously poured into a cuvette maintained at a temperature of 25 °C and subsequently placed within the sample tank for measurement.

### 3.9. Contact Angle

According to the sessile drop method, a **2/3SiDDGPBH** solution with a concentration of 2 g/L was prepared using Ultra-Pure Water (UPW). Under constant temperature and humidity conditions (25 °C, 50% RH), various substrate materials were cut into 3×3 cm pieces. The static water contact angles on the surfaces of the untreated substrates were measured using a fully automated drop shape analyzer. The aqueous solution of the product was then sprayed onto the material surfaces for modification, and the contact angles were remeasured after air drying. To evaluate the long-term stability of the modification effect, the treated samples were subjected to repeated wiping cycles, and the variation trend of the contact angle was further examined.

### 3.10. Scanning Electron Microscopy (SEM)

Initially, fragments measuring approximately 0.5 × 0.5 cm were meticulously extracted using a saw. Given that wood is not conductive, it must be sprayed with gold to minimize charging. Subsequently, the specimen was affixed to the sample stage with conductive adhesive to ensure optimal contact. Subsequently, the specimen was positioned within the sample chamber of the electron microscope, and the requisite vacuum was applied. The optimal accelerating voltage and working distance were then selected. Subsequently, adjustments were made to the focal length and contrast, and a meticulous search was conducted for the target area at low magnification. Subsequently, the target area was systematically expanded to facilitate the observation and acquisition of high-definition images.

## 4. Conclusions

D-gluconic acid δ-lactone was amidated with N, N-dimethyldipropenyltriamine to obtain the glucosylamide precursor (**DDGPD**). **DDGPD** was then reacted with dodecyl bromide to introduce a hydrophobic chain, thereby obtaining the intermediate (**DDGPDH**). The final intermediate was quaternized with γ-chloropropylmethyldimethoxysilane/γ-chloropropyltrimethoxysilane, in synthesizing two kinds of glucosylamide organosilicon quaternary ammonium salts. This synthetic route is concise, efficient, generates no waste, and meets green chemistry principles. The structures of all reaction materials, intermediates, and target products were confirmed by FT-IR, ^1^H NMR, and ^13^C NMR spectroscopy. The surface activity, adsorption and aggregation behavior, and foam properties of **2/3SiDDGPBH** in aqueous solution were investigated by equilibrium surface tension, dynamic surface tension, dynamic light scattering, transmission electron microscopy, and foam analyzer. Furthermore, the surface spreadability of the product-modified materials was examined, and the long-term stability of the modified materials was validated. The two products demonstrated satisfactory surface activity, with critical micelle concentration (CMC) values of **3SiDDGPBH** > **2SiDDGPBH**. These values suggest the potential for reducing the surface tension of water to 33.64 mN/m and 33.4 mN/m, respectively. TEM and DLS analysis revealed that **2/3SiDDGPBH** formed homogeneous spherical micelles with size distributions ranging from 1–10 nm to 30–100 nm. In consideration of the properties of the foam, it was determined that **3SiDDGPBH** exhibited superior capabilities in terms of foaming and stabilizing properties when compared with **2SiDDGPBH**. In the context of material modification, the aqueous solution of **2/3SiDDGPBH** was applied to PMMA board, mature acacia leaves, oil paper, vegetable-tanned head layer cowhide, MFR plywood. The materials exhibited excellent spreading performance and demonstrated long-lasting effects on mature acacia leaves, oil paper, vegetable-tanned head layer cowhide, and MFR plywood. Notably, on MFR plywood, the contact angle remained at 75° and 65° after 80 cycles of scrubbing, indicating the product’s effectiveness and longevity. SEM further demonstrated that a significant quantity of the product remained affixed to the MFR plywood after 80 cycles of scrubbing, indicating that the product exhibited a strong chemical bond with the MFR plywood, rather than merely physical adsorption. Consequently, this product constitutes a novel class of sugar-based cationic surfactants. Its low surface tension, foaming properties, superior spreading properties, and long-lasting effects are anticipated to have extensive application prospects in areas such as pesticide formulations, industrial and household cleaning products, personal care products, oil and gas extraction, textile processing, and specialized coatings.

## Figures and Tables

**Table 1:** Surface property parameters of the **2/3SiDDGPBH** in aqueous solutions.

Surfactant	*CMC* (g/L)	*γ_cmc_* (mN/m)	*Γ_max_* (mol·cm^−2^)	*A_min_* (Å^2^)	$\Delta G_{ads}^{ϴ}$ (kJ/mol)	$\Delta G_{mic}^{ϴ}$ (kJ/mol)
**2SiDDGPBH**	0.203	33.4	8.27 × 10^−11^	200.91	−81.09	−25.89
**3SiDDGPBH**	0.285	33.64	7.95 × 10^−11^	208.87	−80.99	−25.15

## Data Availability

The original contributions presented in this study are included in the article/Supplementary Materials. Further inquiries can be directed to the corresponding author.

## Supplementary Materials

The following supporting information can be downloaded at https://www.mdpi.com/article/10.3390/molecules30193934/s1, Figure S1: ^1^H-NMR spectra of **DDGPD**; Figure S2: ^1^H-NMR spectra of **DDGPDH**; Figure S3: ^1^H-NMR spectra of **2SiDDGPBH**; Figure S4: ^13^C-NMR spectra of **DDGPD**; Figure S5: ^13^C-NMR spectra of **DDGPDH**; Figure S6: ^13^C-NMR spectra of 2SiDDGPBH; Figure S7: Foam photographs and distribution of **2/3SiDDGPBH** at different concentrations; Figure S8: Foam size distribution histogram of **2/3SiDDGPBH** at different concentrations.
