# Supplementary material for: The Synthesis of Novel Glucosylamide Organosilicon Quaternary Ammonium Salts and Long-Lasting Modification of Different Materials
Source: Molecules. 2025 Oct 1;30(19):3934. doi: 10.3390/molecules30193934 (PMC12525607; doi:10.3390/molecules30193934)
Supplement: Supplementary file 1 [file molecules-30-03934-s001.zip › molecules-3867480-supplementary.pdf]

Supplementary materials

The Synthesis of Novel Glucosylamide Organosilicon Quaternary Ammonium Salts and Long-Lasting Modification of Different Materials

Xiangji Meng [1], Yunkai Wang [1], Jingru Wang [1], Lifei Zhi [1,*], Linfei Li [1], Xiaoming Li [1], Chan Wu [1], Rui Jin [2], Ziyong Ma [3], Zhiwang Han [1] and Xudong Liu [4]

[1] College of Chemical Engineering and Technology, Taiyuan University of Science and Technology, Taiyuan 030024, China; s202321111068@stu.tyust.edu.cn (X.M.); wyk990306@163.com (Y.W.); s202321111083@stu.tyust.edu.cn (J.W.); lilinfei@tyust.edu.cn (L.L.); xiaomingli@tyust.edu.cn (X.L.); 202421020224@stu.tyust.edu.cn (C.W.); hahzw@tyust.edu.cn (Z.H.)
[2] Jiangsu Wanqi Biotechnology Co., Ltd., Hai'an 226600; jinrui@wanqicn.net
[3] Hai'an R&D Center of High-End Equipment and Rail Transit, Taiyuan University of Science and Technology, Hai'an 226600; zyma_sc@tyust.edu.cn
[4] Taiyuan Hengdeyuan Animal Health Care Technology R&D Co., Ltd., Taiyuan 030024, China; 13835179079@163.com
* Correspondence: lifeizhi@yeah.net or lifeizhi@tyust.edu.cn

Table of Content

(1) NMR hydrogen spectra of DDGPD, DDGPDH and 2SiDDGPBH

(2) NMR carbon spectra of DDGPD, DDGPDH and 2SiDDGPBH

(3) The foam structure and distribution of 0.1g/L versus 1g/L

1. NMR hydrogen spectra of DDGPD, DDGPDH and 2SiDDGPBH are shown in Figures S1-S3

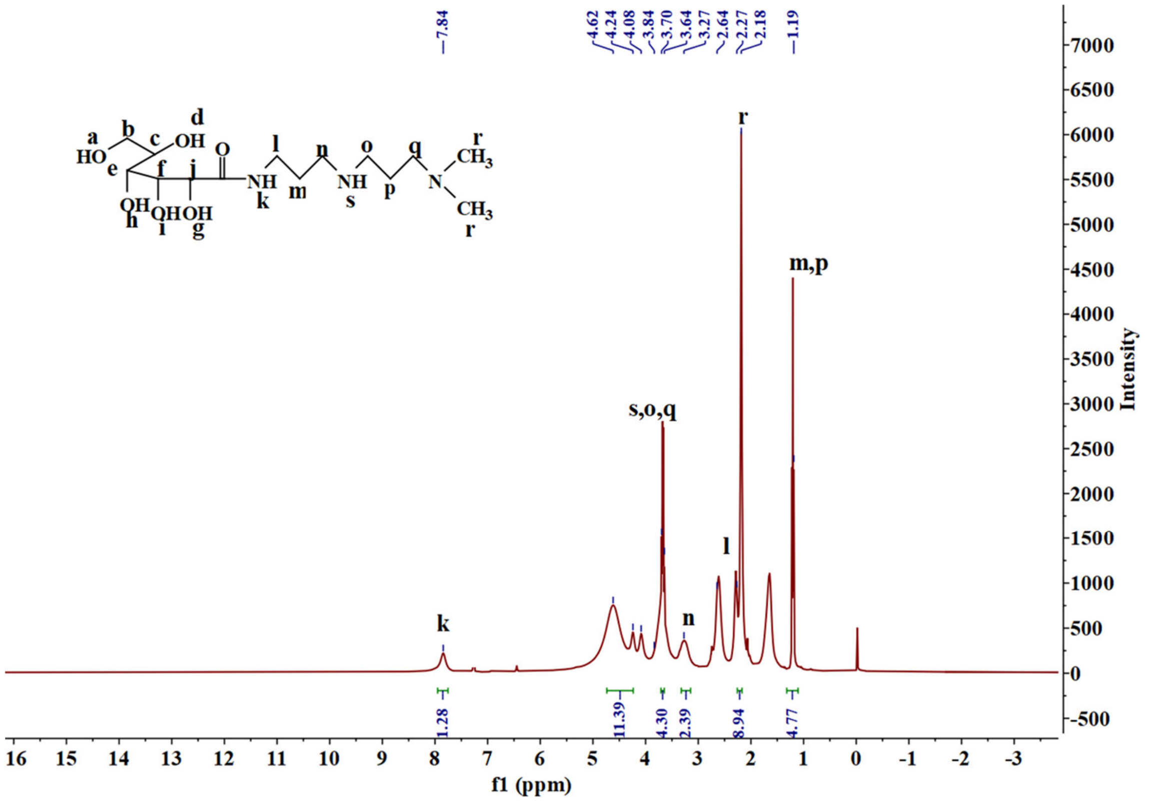

Figure S1. ^{1}H-NMR spectra of DDGPD

DDGPD : ^{1}H-NMR(DMSO-d6, 600MHz): δ : 1.19 (m, 4H, $NHCH_2C\underline{\mathbf{H_2}}$, $NHCH_2C\underline{\mathbf{H_2}}CH_2$), 2.18 (s, 6H, $NC\underline{\mathbf{H_3}}C\underline{\mathbf{H_3}}$), 2.27 (t, 2H, $NHCH_2CH_2C\underline{\mathbf{H_2}}NHCO$), 3.27 (t, 2H, $NHC\mathbf{H_2}CH_2CH_2$ NHCO), 3.64 (s, 1H, $N\underline{\mathbf{H}}$), 3.70 (m, 2H, $NHC\underline{\mathbf{H_2}}CH_2CH_2N(CH_3)_2$), 3.84 (m, 2H, $NHCH_2\,CH_2C\underline{\mathbf{H_2}}N(CH_3)_2$), 3.84-4.62 (m, 11H, O**H** groups from sugar part), 7.84 (s, 1H, CON**H**).

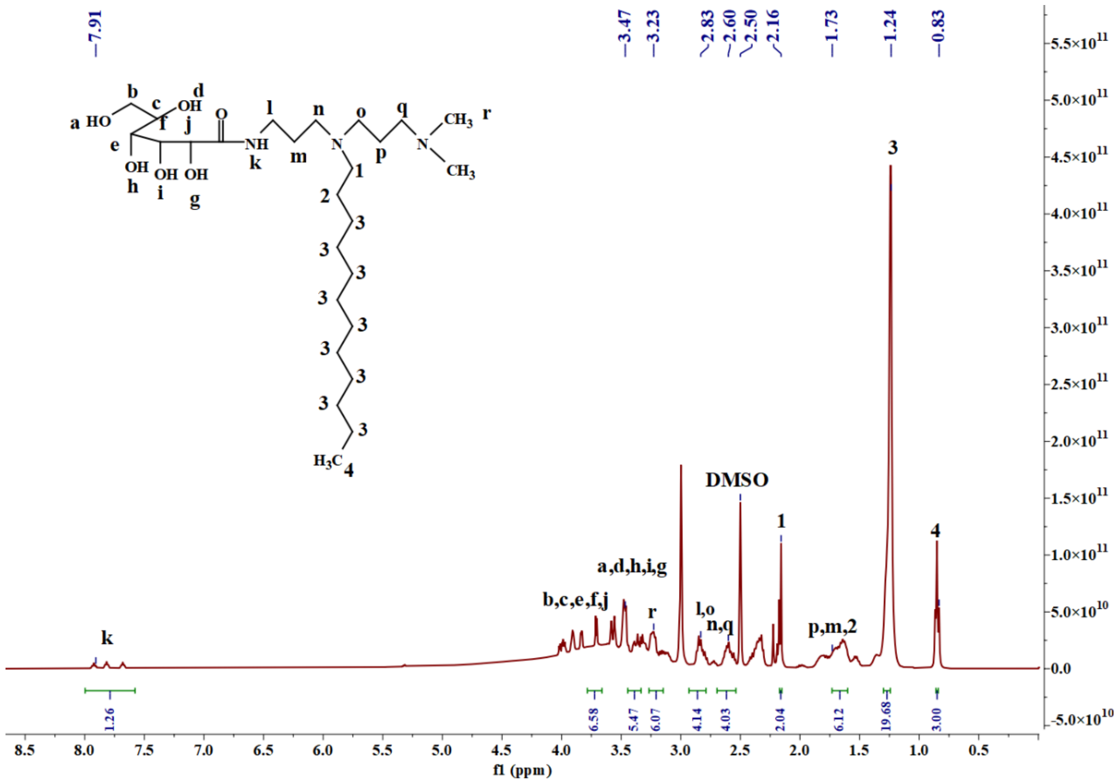

Figure S2. ^{1}H-NMR spectra of DDGPDH

DDGPDH : ^{1}H-NMR(DMSO-d6, 600MHz): δ: 0.83 (m, 3H, $CH_2CH_2(CH_2)_9C\mathbf{H_3}$), 1.24 (s, 18H, $CH_2CH_2(C\underline{\mathbf{H_2}})_9CH_3$), 1.73 (m, 6H, $NCH_2C\underline{\mathbf{H_2}}CH_2$, $NCH_2C\underline{\mathbf{H_2}}CH_2$, $NCH_2C\underline{\mathbf{H_2}}(CH_2)_9CH_3$), 2.16 (m, 2H, $NC\underline{\mathbf{H_2}}CH_2(CH_2)_9CH_3$), 2.60 (m, 4H, $NCH_2CH_2C\underline{\mathbf{H_2}}$, $NCH_2CH_2C\underline{\mathbf{H_2}}$), 2.83 (m, 4H, $NC\underline{\mathbf{H_2}}CH_2CH_2$, $NC\underline{\mathbf{H_2}}CH_2CH_2$), 3.23 (d, 6H, $NC\underline{\mathbf{H_3}}$, $NC\underline{\mathbf{H_3}}$), 3.47 (m, 5H, $CONHC\underline{\mathbf{H_2}}$, $NC\underline{\mathbf{H_2}}CH_2CH_2N$, $O\underline{\mathbf{H}}$ groups from sugar part), 3.62-4.28 (m, 6H, $O\underline{\mathbf{H}}$ groups from sugar part), 7.91(d, 1H, $CON\underline{\mathbf{H}}$).

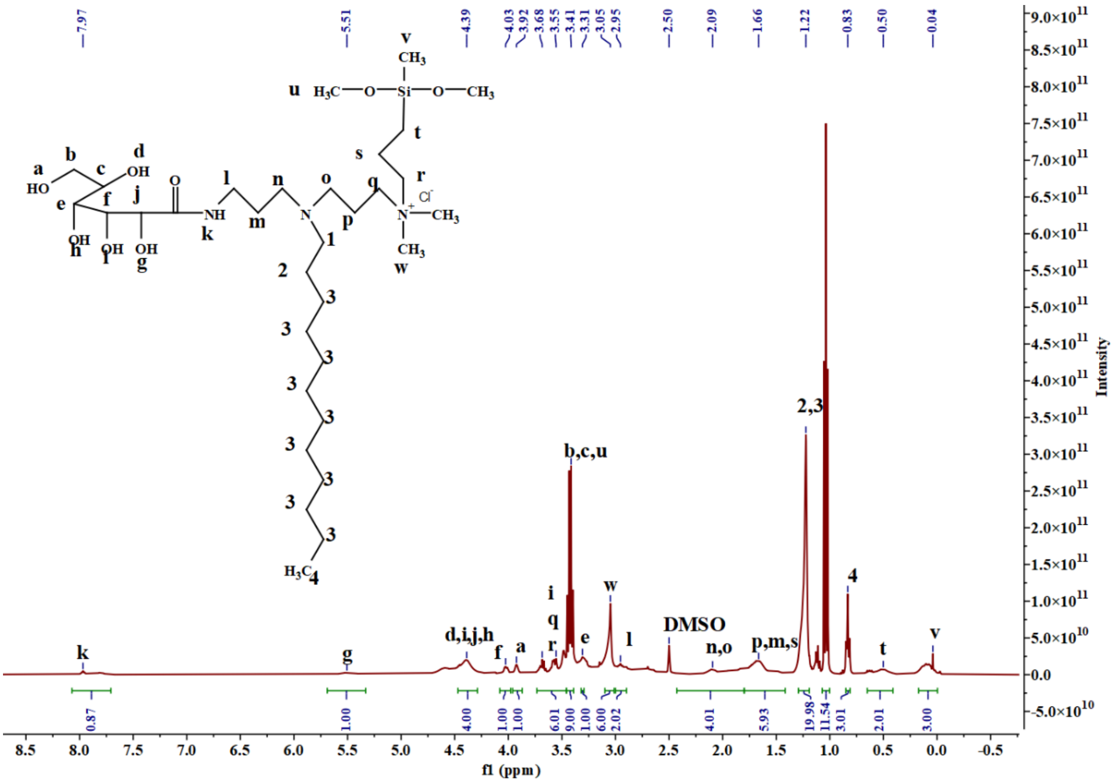

Figure S3. ^{1}H-NMR spectra of 2SiDDGPBH

2SiDDGPBH : ^{1}H-NMR(DMSO-d6, ppm): δ : 0.04 (m,3H,$SiC\underline{\mathbf{H_3}}$), 0.50 (d, 2H, $SiC\underline{\mathbf{H_2}}$), 0.83 (t, 3H, $NCH_2(CH_2)_{10}C\underline{\mathbf{H_3}}$), 1.22 (s, 20H, $NCH_2\underline{\mathbf{(CH_2)_5}}$CH3), 1.66 (t, 6H, $NCH_2C\underline{\mathbf{H_2}}CH_2N^+$, $NCH_2C\underline{\mathbf{H_2}}CH_2N$, $SiCH_2C\underline{\mathbf{H_2}}$), 2.09 (s, 4H, $NC\underline{\mathbf{H_2}}$ CH_2,$NC\underline{\mathbf{H}}_2CH_2$), 2.95 (m, 2H, $NC\underline{\mathbf{H}}_2$), 3.05 (s, 6H,$N^+C\underline{\mathbf{H}}_3C\underline{\mathbf{H}}_3$), 3.31 (s, 1H, COCH(OH)CH(OH)CH(O$\underline{\mathbf{H}}$), 3.41 (m, 9H, $Si(OC\underline{\mathbf{H}}_3)_2$, $COCH(OH)_3C\underline{\mathbf{H}}OHC\underline{\mathbf{H}}_2OH$),3.55 (t, 2H, $N^+C\underline{\mathbf{H}}_2$), 3.68 (m, 4H, $N(CH_2CH_2C\underline{\mathbf{H}}_2)_2$, 3.92 (s, 1H, $COCH(OH)_3CHOHCH_2O\underline{\mathbf{H}}$), 4.03 (d, 1H, COCH(OH)C$\underline{\mathbf{H}}$(OH)), 4.39 (t, 4H, $COCH(O\underline{\mathbf{H}})_4CH_2O\underline{H}$), 5.51 (d, 1H, COCH(O$\underline{\mathbf{H}}$), 7.97 (s, 1H, CON$\underline{\mathbf{H}}$).

2. NMR carbon spectra of DDGPD, DDGPDH and 2SiDDGPBH are shown in Figures S4-S6.

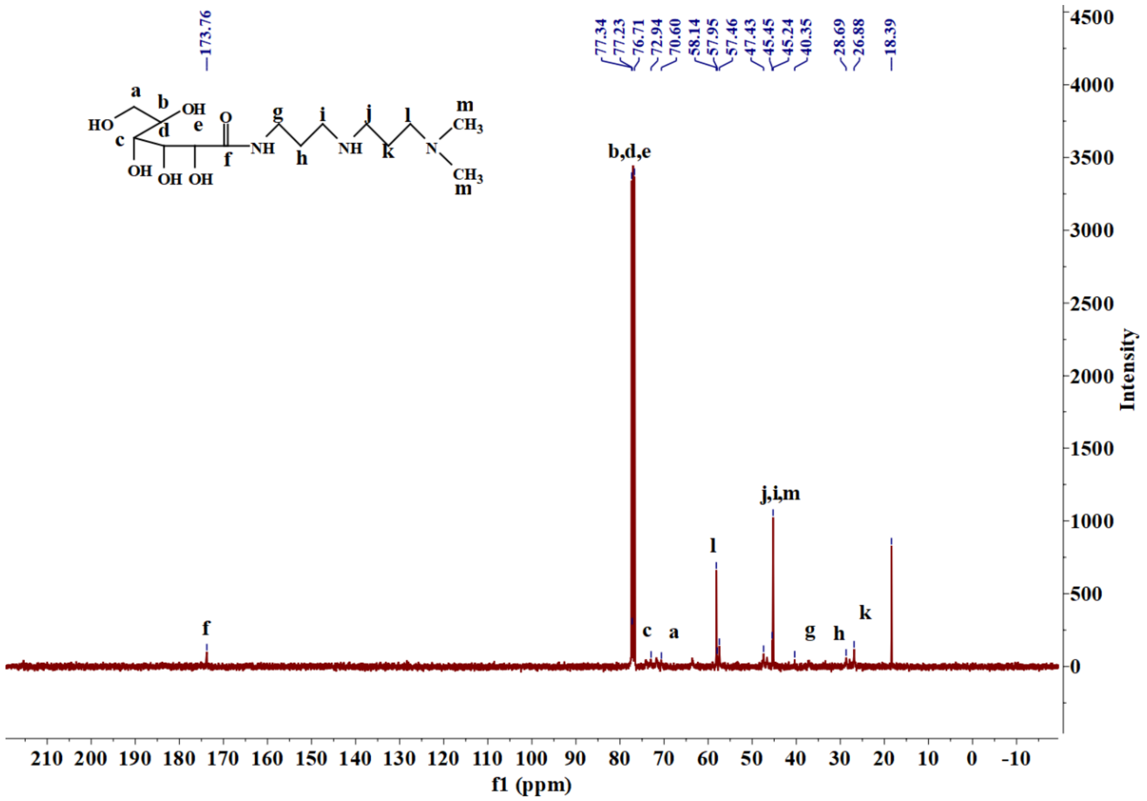

Figure S4. ^{13}C-NMR spectra of DDGPD

DDGPD : ^{13}C-NMR(DMSO-d6, 600MHz): δ: 26.88 ($NHCH_2\underline{\mathbf{C}}H_2CH_2N$), 28.69 ($CONHCH_2\underline{\mathbf{C}}H_2CH_2NH$), 40.35 ($CONH\underline{\mathbf{C}}H_2CH_2CH_2NH$), 45.24 ($N\underline{\mathbf{C}}H_3$, $N\underline{\mathbf{C}}H_3$), 45.45 ($CONHCH_2CH_2\underline{\mathbf{C}}H_2NH$), 47.43($NH\underline{\mathbf{C}}H_2CH_2CH_2N$), 57.46 ($NH\underline{C}H_2CH_2\mathbf{C}H_2N$), 57.95 ($NHCH_2CH_2\mathbf{C}H_2N\underline{\mathbf{C}}H_3$, $NHCH_2CH_2\mathbf{C}H_2N\underline{\mathbf{C}}H_3$),58.14 ($NHCH_2CH_2\underline{\mathbf{C}}H_2N$), 70.60 ($\underline{\mathbf{C}}HOH$), 72.94 ($\underline{\mathbf{C}}HOH$), 76.71 ($\underline{\mathbf{C}}HOH$), 77.23 ($\underline{\mathbf{C}}HOH$), 77.34 ($\underline{\mathbf{C}}HOH$), 173.76 ($\underline{\mathbf{C}}ONH$).

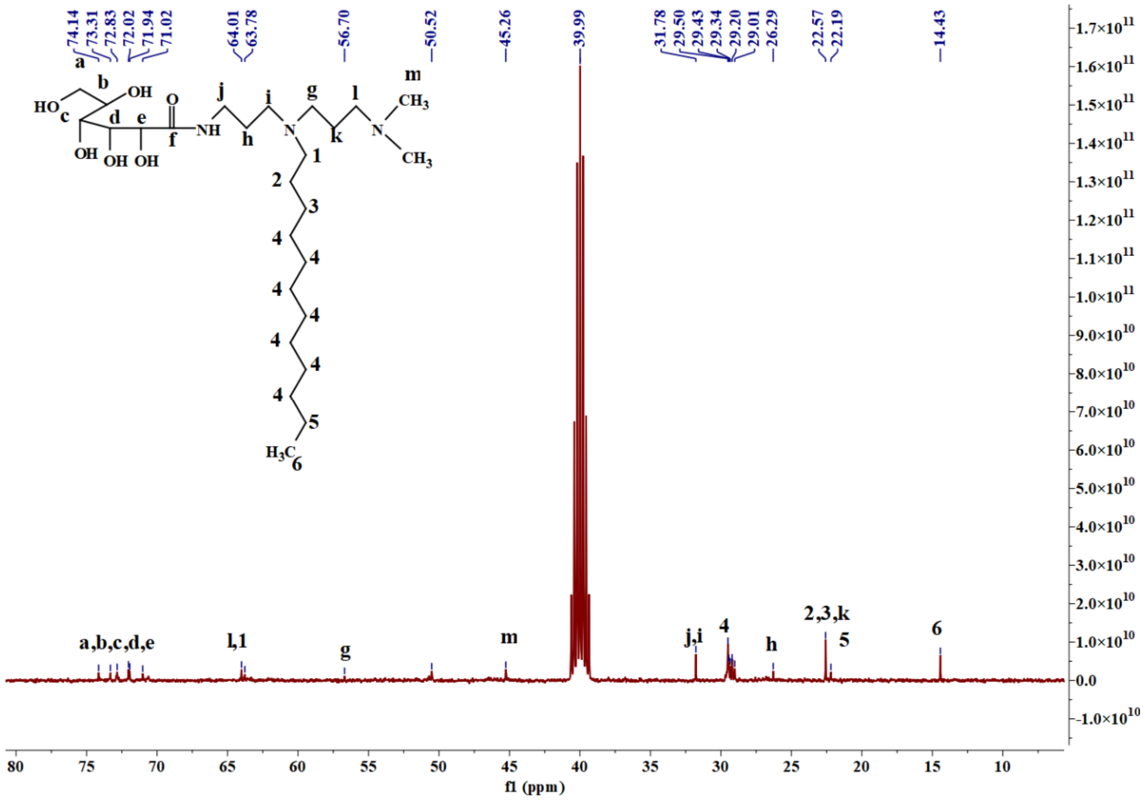

Figure S5. ^{13}C-NMR spectra of DDGPDH

DDGPDH : ^{13}C-NMR(DMSO-d6, 600MHz): δ: 14.43 ($CH_2CH_2(CH_2)_{11}\underline{C}H_3$, $CH_2CH_2(CH_2)_{11}\underline{C}H_3$), 22.19 ($N(CH_2)_{10}\underline{C}H_2CH_3$), 22.57 ($N^+(CH_2)_{10}\underline{C}H_2CH_3$, $N(CH_2)_4(\underline{C}H_2)_{\underline{8}}CH_2CH_3$, $N^+(CH_2)_4(\underline{C}H_2)_{\underline{8}}CH_2CH_3$), 26.29 ($N(CH_2)_3\underline{C}H_2(CH_2)_9CH_3$, $N^+(CH_2)_3\ \underline{C}H_2(CH_2)_9CH_3$), 27.97 ($N^+(CH_2)_2\underline{C}H_2(CH_2)_{10}CH_3$), 29.01 ($N(CH_2)_2\underline{C}H_2(CH_2)_{10}CH_3$), 29.20 ($NCH_2\underline{C}H_2CH_2N^+$), 29.34($CONHCH_2\underline{C}H_2CH_2N$), 29.43 ($NCH_2\underline{C}H_2(CH_2)_{11}CH_3$), 29.50 ($N^+CH_2\underline{C}H_2(CH_2)_{11}CH_3$), 31.78 ($CONH\underline{C}H_2CH_2CH_2N$), 45.26 ($N\underline{C}H_2(CH_2)_{12}CH_3$, $CONHCH_2CH_2\underline{C}H_2N$), 50.52 ($N\underline{C}H_2CH_2CH_2N^+$), 63.78 ($N^+CH_3$, N^+CH_3), 64.01 $NCH_2CH_2\underline{C}H_2N^+$, $N^+\underline{C}H_2(CH_2)_{12}CH_3$ (), 71.02 ($\underline{C}H_2OH$), 71.94 ($\underline{C}H_2OH$), 72.02 ($\underline{C}HOH$), 72.83 ($\underline{C}HOH$), 73.31 ($\underline{C}HOH, \underline{C}HOH, \underline{C}HOH, \underline{C}HOH$), 74.14 ($\underline{C}HO,\underline{C}HO,O\underline{C}HO$), 173.78 ($\underline{C}ONH$).

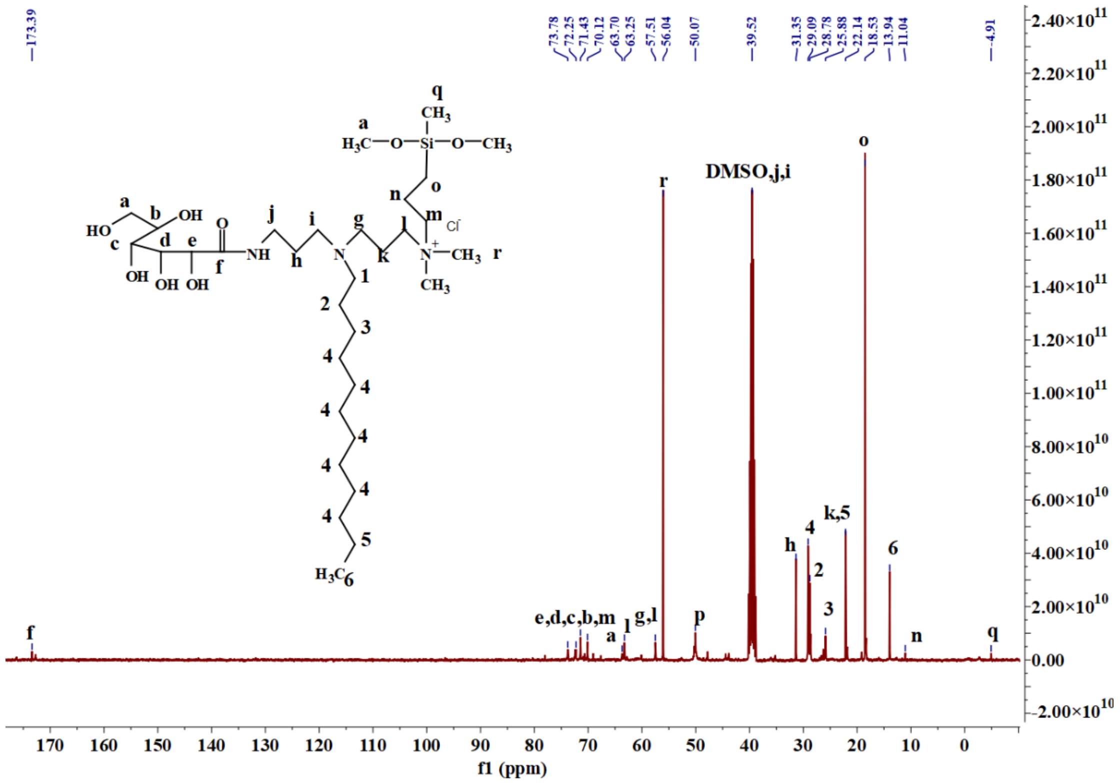

Figure S6. ^{13}C-NMR spectra of 2SiDDGPBH

2SiDDGPBH : ^{13}C-NMR(DMSO ,ppm): -4.91 ($Si\underline{C}H_3$), 11.04 ($N^+CH_2\underline{C}H_2$), 13.94 ($N(CH_2)_{11}\underline{C}H_3$), 18.53 ($Si\underline{C}H_2$), 22.14 ($N(CH_2)_{10}\underline{C}H_2$, $NCH_2\underline{C}H_2CH_2N^+$), 25.88 ($N(CH_2)_2\underline{C}H_2$), 28.78 ($NCH_2\underline{C}H_2$), 29.09 ($N(CH_2)_3(\underline{C}H_2)_7$, 31.35 ($NCH_2\underline{C}H_2CH_2NH$), 39.52 ($N\underline{C}H_2CH_2\underline{C}H_2NH$), 50.07 ($SiO(\underline{C}H_3)_2$), 56.04 ($N^+(\underline{C}H_3)_2$), 57.51 ($N\underline{C}H_2CH_2\underline{C}H_2N^+$), 63.25 ($NCH_2(CH_2)_{10}CH_3$), 63.70 ($COCHOHCHOHCHOHCHOH\underline{C}H_2OH$), 70.12 ($N^+\underline{C}H_2$), 71.43 ($COCHOHCHOH\underline{C}HOH\underline{C}HOHCH_2OH$), 72.25 ($COCHOH\underline{C}HOHCHOHCHOHCH_2OH$), 73.78 ($CO\underline{C}HOHCHOH\underline{C}HOH\underline{C}HOHCH_2OH$).

3. The foam structure and distribution of 3.0.1g/L versus 1g/L is demonstrated in Figures S7-S8.

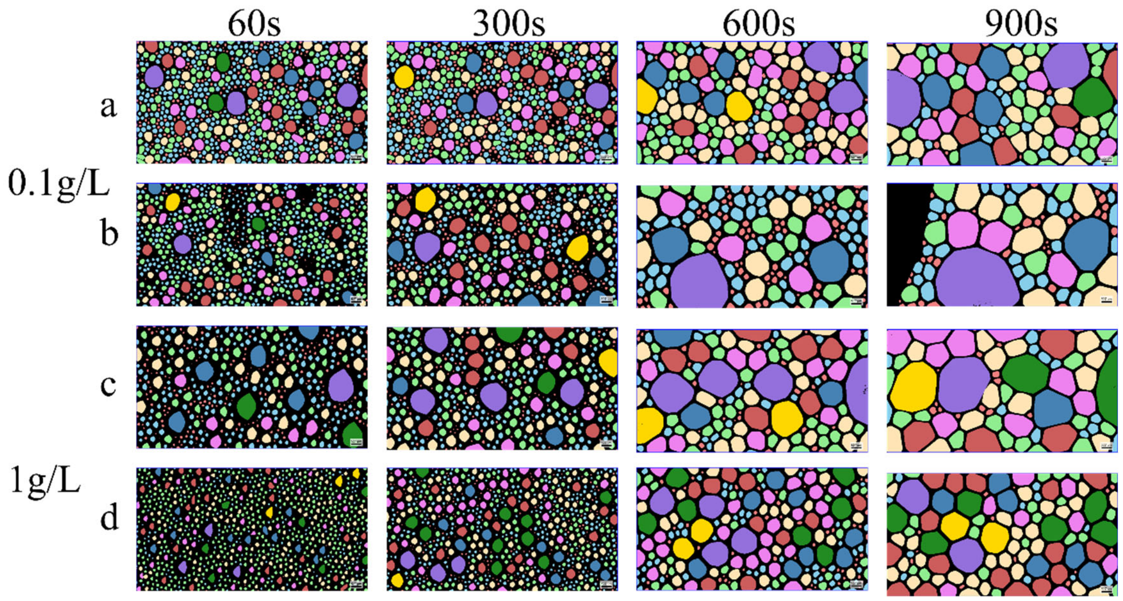

Figure S7. Foam photographs and distribution of 2/3SiDDGPBH at different concentrations. (a)0.1 g/L 2SiDDGPBH, (b)0.1 g/L 3SiDDGPBH, (c)1 g/L 2SiDDGPBH, (d) 1 g/L 3SiDDGPBH

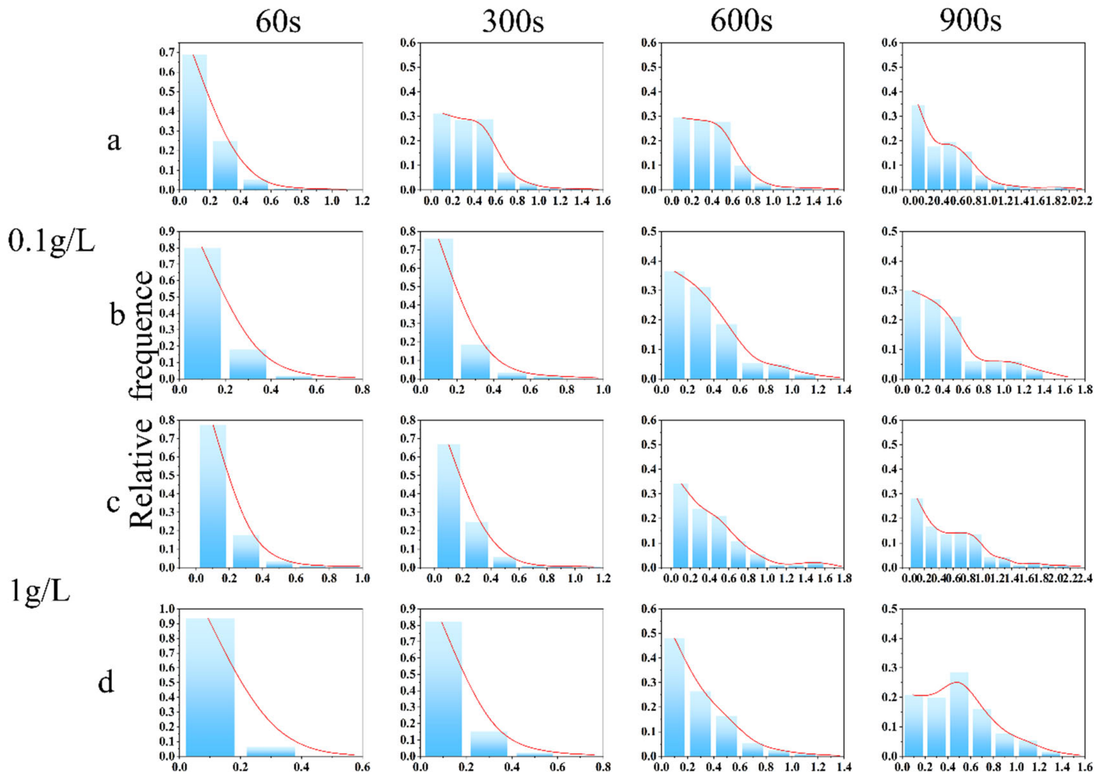

Figure S8. Foam size distribution histogram of 2/3SiDDGPBH at different concentrations. (a)0.1 g/L 2SiDDGPBH, (b)0.1 g/L 3SiDDGPBH, (c)1 g/L 2SiDDGPBH, (d) 1 g/L 3SiDDGPBH
